# Surgical Club of South West England—Meeting in Barnstaple, Oct. 1988

**Published:** 1989-08

**Authors:** 


### Surgical Club of S. W. England

### Meeting at Barnstaple, October 1988

### ADJUVANT CHEMOTHERAPY IN EARLY BREAST CARCINOMA

**J R Barker**
North Devon District Hospital, Barnstaple

A study was undertaken of the patients with early breast carcinoma treated by the author between the years 1976 and 1986. Those patients who were premenapausal were treated with Simple Mastectomy or Wide Local Excision and cytotoxic drugs; the postmenopausal patients were given the same surgical treatment but received Tamoxifen long term after the operation. The cytotoxic drugs used were Thiotepa 30 mg., Vincristine 2 mg and Fluorouracil 250 mg intravenously immediately after the operation and three further doses at three weekly intervals.

There were 220 patients in all, 87 received cytotoxics and 133 Tamoxifen. The age of the patients varied from 31 to 91 with an average of 62.5 years. All completed the course of cytotoxics but 17 or 19% with a reduced dose because of the haematological effects. No patient required a wig although a few complained of slight thinning of their hair. Three developed pins and needles in the fingers but this resolved spontaneously within three months.

The overall survival was 78% at 5 years and 46% at 10 years. In those receiving cytotoxic drugs 84% were alive at 5 years as against 77% in those taking Tamoxifen. At 10 years 75% of the cytotoxic group were alive as against 38% of the Tamoxifen group. In those patients with T 1&2 tumours, 90% were alive at 5 years and 85% at 10 years in the Cytotoxic group as against 76% at 5 years and 50% at ten years in the Tamoxifen. Unfortunately the numbers were too small to be statically significant.

### COLONIC BOWEL RESECTIONS: NO MECHANICAL BOWEL PREPARATION:

**John R Barker**

A retrospective study of 129 consecative large bowl resections performed by the author between January 1986 and December 1987 was undertaken. All the patients had Ampicillin and Metronidazole preoperatively which was continued for five days. They took a normal diet and were given no form of mechanical bowel preparation.

The average age was 66 years with a range 16–93 years. 90 had carcinoma, 22 Diverticulosis, 10 Crohn's Disease and 6 Ulcerative Colitis. 28% had an ilostomy or colostomy and 72% an anastomosis. 26% were performed as an emergency. Of the 86 who had ananastomosis, 52 or 60% were on the left side. Two layers of continous catgut was used for the anastomoses, and an anal dilation performed at the end of the operation.

There were three deaths, 2.3%, one from ischeamia of the whole gut which followed an anastomotic leak from sigmoid resection for diverticular abcess; one from Congestive Cardiac Failure and one from a Myocardial Infarction. There wer two anastomotic leaks, 2.3%, one as mentioned in the mortality figures and on ilio-rectal anastomosis in a 79 year old with Crohn's Disease which was converted to an ilostomy and survived. 15 Patients, 11%, developed a wound infection 11 due to *E. Coli*, 2 to *Staphlococi* and 2 to *Pseudomonas*; all were sensative to Gentamycin.

These results compare very favourably with those in which bowel preparation is used but the author now gives a single dose of Gentamycin at the completion of the operation.

### OPHTHALMIC TRAUMA

**R A Gibson**
N. Devon District Hospital, Barnstaple

The general surgeon dealing with trauma cases needs some familiarity with ocular injuries to cope with their initial management, although the car seat belt law has significantly reduced their incidence.

Cases of 'blunt' trauma to the eyelids, eyeball and orbit are by far the commonest ophthalmic injury. The 'black eye' needs careful examination to detect injury within the eye, particularly an anterior chamber bleed (hyphaema) or vitreous haemorrhage. Retinal oedema and haemorrhage are sometimes found and rarely a dislocated lens. 'Whiplash injuries' can damage the optic nerves in the orbit or at the chiasm. Orbital trauma can lead to double vision, particularly the orbital floor 'blow-out' fracture with entrapment of inferior orbital tissue in the fracture line.

Perforating trauma to the eyeball is thankfully now rare but is usually obvious on careful eye examination. Loss of the clear corneal and anterior chamber appearance together with an abnormal pupil shape are good pointers to an ocular perforation. A medial eyelid laceration may involve the tear drainage apparatus and an upper lid laceration may involve the levator muscle complex.

Recognition of the ophthalmic injury is the first essential followed by referral if the injury requires direct ophthalmic specialist involvement.

### DEATHS DUE TO MOTOR-CYCLES

**C M D Ross**
N. Devon District Hospital, Barnstaple

A review of 70 traffic-related deaths in North Devon over a 5-year period (1983–1987) showed almost 25% to be riders and passengers on motorcycles. The proportion for Devon as a whole was 21% but motorcycles represent only about 8% of licensed vehicles in the County.

Nationally, the proportion of motorcycles is even less, (about 5%), while motorcyclists account for 15.6% of annual fatalities.

A further disproportion was found in fatalities to pedestrians struck by motorcyclists — nearly 30% of pedestrian fatalities.

The broad nature of injuries differed little from those in other fatalities but the victims were nearly all young males, less than 20 years old. By contrast, pedestrians were preponderantly old women, 60 + years.

A small category of related deaths was identified, termed Post-accident Suicide — the wife of a paraplegic motorcyclist, the (innocent) driver of a car following fatal collision with a motorcycle and an eccentric who ate all the Co-proxamol prescribed for the pain of a fracture sustained in a motorcycling escapade.

The reasons for accidents were adduced as:

(1) The State of the Machine
(2) Capability of the Operator
(3) Road Conditions
(4) Other Road-Users
(5) The nature of the Machine.

It is difficult adequately to regulate the first four of these variables but it was proposed that the motor cycle should be regarded as an epidemidogical threat to be eliminated from society, if necessary, by legislation; albeit with some regret.

### MOTORBIKE LEGS

**Richard L M Newell**
N. Devon District Hospital, Barnstaple

The history and development of the motorcycle and its rise to popularity were related to the potential of two-wheeled vehicles for causing injury to the lower limbs, starting with H G Wells' descriptions in his book 'The Wheels of Chance'. A general review of methods of treatment of leg injuries from classical times to the present day led into the presentation of a consecutive and unselected series of six young motorcyclists, all of whom had very severe lower limb trauma.

All were treated conservatively according to basic surgical principles: only in one case was minimal internal fixation used. The most severely injured patient had a secondary above-knee amputation, while the others all regained mobile knees with full bone and soft-tissue healing. All six walk unaided, and five are working.

### ASYMPTOMATIC AND RECURRENT PARATHYROIDISM

**J. R. Farndon.**
Bristol Royal Infirmary
Printed in extenso on page 70.

### Registrar's Prize Papers

### INTRA-OPERATIVE BREAST CYTOLOGY: AN AID FOR ASSESSING EXTENT OF CONSERVATIVE SURGERY

**Lucarotti Michele, White H, (Leaper D J)**
University Departments of Surgery and Pathology, Southmead Hospital, Bristol

Limited surgery for breast cancer cannot ensure that the cancer is adequately excised. There are no guidelines for the extent of surrounding normal breast tissue which should be included.

We have examined post-excision cavities intra-operatively using cytology to accurately assess whether excision is complete in 81 patients undergoing breast lump excision for suspected or confirmed carcinoma. Cytological scrapings were taken from the excision cavity immediately prior to skin closure and air-dried slides prepared. Fifty patients had a final diagnosis of carcinoma and 31 had benign disease.

Ten patients with malignant cell smears had histological evidence of incomplete excision, lymphatic or vascular invasion. Seven patients with incomplete histological excision had benign cells only on cytology. There were no false positives in the completely excised group (36) or the benign group (31). This discrimination was statistically significant (Yates $\times^2$ $p<0.01$).

Cell cultures were grown from three of the tumours and the effects of 8 antiseptic or antitumour topical agents investigated. Chlorhexidine 5% was found to be the most cytotoxic (90% cytotoxicity at 1:10,000 strength).

We suggest that intra-operative cytology of the cavity after conservative excision of a breast cancer may be a useful adjunct to ensure the accuracy of surgery and that antiseptic irrigation may help to prevent local recurrence.

### REVIEW OF PATIENTS ON A WAITING LIST WITH PROSTATIC SYMPTOMS

**C P Barham E D James, R D Pocock**
Royal Devon & Exeter Hospital

Ninety patients awaiting surgery for bladder outflow obstruction were reassessed with regard to their symptoms, the results of their peak urinary flow rates, measurement of urinary residuals by ultrasound examination and a frequency/volume chart.

Symptoms had improved in eleven patients, were worse in twenty, while fifty-six remained unchanged. There was no significant difference between the waiting length time of these three groups. Two patients had developed acute retention and one patient had died.

Following reassessment, thirty-nine patients were removed from the waiting list. Six had no evidence of obstruction. Thirty had evidence of obstruction, but their symptoms were minor, they had small urinary residuals and they were reluctant to have surgery. A further twenty patients were removed from the waiting list but kept under review because of symptoms of bladder instability or because they had minor symptoms, near normal flow rates but large residuals. Only thirty-one patients were kept on the waiting list and six of these were expedited because of severe obstructive symptoms.

This study re-emphasizes the importance of objective tests on patients with obstructive symptoms. It confirms that progression of symptoms and the development of acute retention is rare. Many patients can be managed conservatively if reassured about the benign nature of their condition and told their symptoms are unlikely to get worse.

### HEPATITIS, AIDS AND THE SURGEON'S LEFT HAND: A STUDY OF THE MECHANISMS AND SITE OF SURGICAL GLOVE PERFORATION

**J D Palmer, J W S Rickett**
Torbay Hospital

Intact surgical gloves are a barrier to Hepatitis B virus and HIV but once perforated during surgery they can not sustain adequate defence. The study examines the rate of glove performations during surgery in a District General Hospital. 275 paris of gloves were collected from 100 consecutive operations. From the 43% of gloves that had been damaged 200 perforations were recorded at a mean rate of 1.18 perforations per operation. Most injuries were to the non-dominant index and middle fingers and occurred during manipulation of the suture needles particularly at wound closure. Using current data relating to Hepatitis B and HIV incidence we estimate that in the lifetime of a general surgeon he may expect to have 3 infections from Hepatitis B and a 0.135% risk of HIV infection. With these results in mind methods of reducing the risk to operating theatre staff are discussed.

